# Selected Abstracts from the 26th Annual Meeting of the Society in Europe for Simulation Applied to Medicine: Virtual. 14-16 April 2021

**DOI:** 10.1186/s41077-021-00164-2

**Published:** 2021-04-12

## A1 Cardiopulmonary resuscitation skills self-training with a novel automated-feedback device: impact in compressions performance

### Topic: Assessment

#### Abel Nicolau^1,2^, Carla Sá-Couto^1,2^, Isabel Sousa^3^, Pedro Vieira-Marques^2,3^

##### ^1^Biomedical Simulation Center, Faculty of Medicine of University of Porto, Portugal; ^2^Center for Health Technology and Services Research (CINTESIS), Portugal; ^3^Informatics Service, Faculty of Medicine of University of Porto, Portugal

###### **Correspondence:** Carla Sá-Couto (csacouto@med.up.pt)

**Introduction**

Cardiopulmonary resuscitation (CPR) is a vital action that may double or quadruple the survival rate from cardiac arrest. Chest compressions are a basilar component of CPR and should be performed with high quality to improve patient outcomes. CPR training promotes acquisition and maintenance of fundamental skills, although it can be time consuming and expensive. To overcome these limitations, several devices are available with automated feedback on the main components of compressions, including frequency, depth, hands positioning and chest recoil. The aim of this work is to study the impact of CPR self-training using a novel automated-feedback device (CPR Personal Trainer) in the compressions performance of healthcare professionals and students. A secondary objective was to evaluate the adherence of the target samples to self-training.

**Methods**

An experimental pre-post study was implemented, with a convenience sample constituted by voluntary medical students (MSt) from the Faculty of Medicine of the University of Porto and medical doctors (MD) and registered nurses (RN) from the Intermediate Care Unit of the University Hospital Center of São João. Ethical approval was obtained prior to the study. CPR Personal Trainer was made available for a 6-week self-training period at the participants work/study place allowing an easy access during shifts/classes. Before the study, all participants had a familiarization session with the device and were advised to train whenever they wish. Before and after the self-training period each participant performed 2 minutes of chest compressions in the device for performance assessment, namely frequency, depth, hands positioning and chest recoil. During the self-training period, the number of trainings, number of compressions and training time were recorded by the system. Statistical analysis was conducted using the IBM SPSS Statistics® software.

**Results & Discussion**

Data was collected from 46 individuals: 12 MD, 17 RN, and 17 MSt. During the self-training period participants spent, in total, 270 minutes using the device. Students’ participation was markedly higher than healthcare professionals, averaging 12.2 training sessions versus 3.2 and 1.6 for RNs and MDs, respectively. Compressions performance scores (Table 1) showed improvements in all components, for all groups, with the exception of chest recoil. Of notice is that, for hands positioning, frequency and depth, the mean values of the post-tests are all within the recommended guidelines, for all groups. Inter-group comparisons showed statistical significant differences in two components before the self-training, but no significant differences after.

This indicates similar performance levels in all groups, after the self-training period. CPR self-training with automated-feedback devices seems to be an adequate strategy for acquisition and maintenance of skills. Further investigation should explore the retention of these gains.

**Acknowledgements**

This works was supported by National Funds through FCT - *Fundação para a Ciência e a Tecnologia* within CINTESIS, R & D Unit (reference UID/IC/4255/2019).

**Ethic Statement**

The authors declare that all procedures followed were in accordance with the ethical standards of the responsible committee on human experimentation (institutional and national) and with the Helsinki Declaration of 1975 (In its most recently amended version). Informed consent was obtained from all patients/participants included in the study.

**Table 1 (abstract A1).** Pre and post test scores for chest compressions components (Mean±SD)

		Pre-test	Post-test	*p*
Hands Positioning (%)	MD	82.7 ± 38.3	98.8 ± 4.0	0.055
	RN	87.0 ± 32.3	100.0 ± 0.0	0.034*
	MSt	88.0 ± 29.9	99.8 ± 1.0	0.069
	*p*	0.999	0.999	
Frequency (cpm)	MD	121.6 ± 11.4	105.5 ± 15.1	0.025*
	RN	140.8 ± 21.9	107.1 ± 25.2	0.001*
	MSt	98.1 ± 18.5	105.9 ± 11.0	0.114
	*p*	<0.001*	0.999	
Depth (cm)	MD	4.8 ± 1.0	5.6 ± 0.6	0.049*
	RN	4.7 ± 0.6	5.2 ± 0.7	0.015*
	MSt	5.8 ± 0.4	5.4 ± 0.6	0.018*
	*p*	<0.001*	0.999	
Chest Recoil (%)	MD	83.6 ± 34.2	67.8 ± 39.4	0.916
	RN	64.8 ± 36.3	82.6 ± 31.6	0.062
	MSt	56.0 ± 38.7	58.2 ± 36.1	0.359
	*p*	0.273	0.267	

* p < 0.05, statistically significant

Tests used: Kruskal-Wallis for independent samples (presented p-values adjusted by Bonferroni correction); Wilcoxon Sign Rank for paired samples, one-tailed

MD – Medical Doctor (n=12); RN – Registred Nurse (17); MSt – Medical Student (n=17)

## A2 Using simulation to assess patient’s self-triage for unscheduled urgent care: the ODISSEE Platform

### Topic: Assessment

#### Allison Gilbert^1^, Edmond Brasseur^1^, Sophie François^2^, Vincent D'Orio^1^, Alexandre Ghuysen^1^

##### ^1^University Hospital of Liège, Belgium; ^2^University of Liège, Belgium

###### **Correspondence:** Allison Gilbert (allison.gilbert@chuliege.be)

**Introduction**

Development of new technologies in the healthcare system is a current and exciting concern for many physicians. Management of unscheduled primary and emergency care is one of the many areas in which technological innovations are starting to emerge.

We developed a French-language self-triage platform, called ODISSEE (*Outil Décisionnel et Informatif des Structures de Soins Efficientes Existantes*), based on previously validated protocols for the triage of out-of-hours primary care calls. We aim to demonstrate its validity and safety as regards patients level of care needs using simulated triage.

**Methods**

**Fig. 1 (abstract A2).**
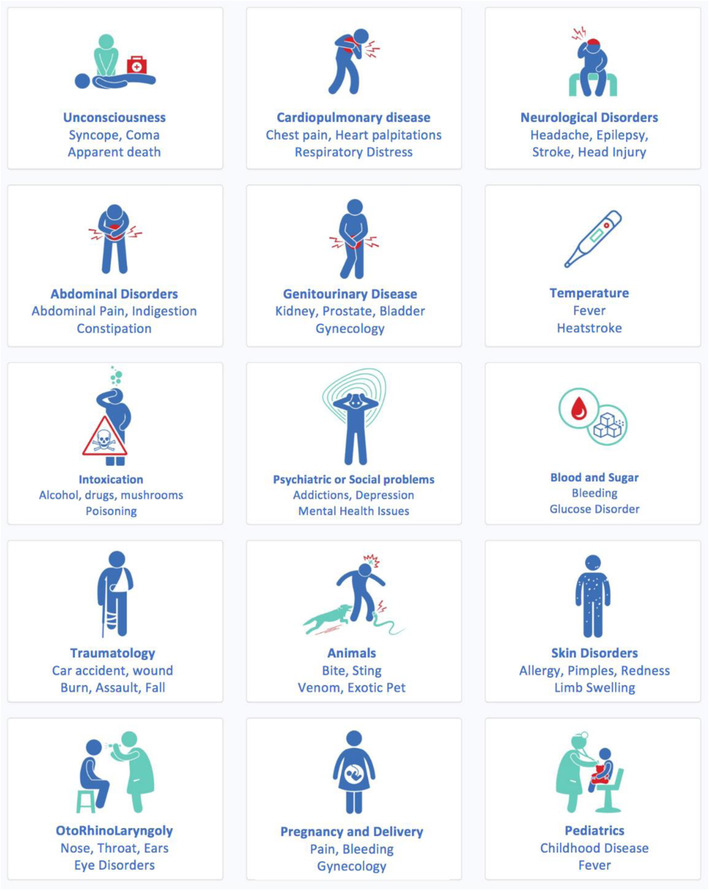
ODISSEE platform

ODISSEE platform is composed of different pictures related to pathologies encountered in unscheduled care settings (Figure 1). Those pictures lead to various algorithmic questionings and, finally, to a theoretical proposition of referral among 4 possibilities: Emergency Medical Services Intervention, Emergency Department referred consultation, Primary care physician immediate or delayed visit.

During a 3-week period, all patients admitted to the Emergency Department of the University Hospital of Liège were eligible to participate to the study, excluding non-native French speakers and patients with an immediate life-threatening condition. Patients were asked to use ODISSEE on a tablet computer to perform the triage simulation. We compared this patient self-assessment with proper nursing triage using the SALOMON algorithm.

**Results & Discussion**

Four hundred and seventeen patients were included into the study. The app was able to find an orientation in 88.2% of the cases (n=368). Among them, 85.1% (n=313) of patients were appropriately triaged. Contrariwise, 14.9% (n=55) of the population was not correctly referred with an over-triage of 9.5% and an under-triage of 5.4%.

Among the participants, 86.8% expressed their satisfaction with the advice given and 80.4% explained they would probably use the platform if she was available. The tool’s performance to predict the need of an Emergency Department referral demonstrated a sensitivity and a specificity of respectively 92.8% and 60%.

Based on these results from our simulation study, we believe that the ODISSEE platform could be a promising technological innovation to safely guide patients in need of unscheduled care to the most appropriate location.

**Ethic Statement**

The authors declare that all procedures followed were in accordance with the ethical standards of the responsible committee on human experimentation (institutional and national) and with the Helsinki Declaration of 1975 (In its most recently amended version). Informed consent was obtained from all patients/participants included in the study.

## A3 Validating a French-language version of Health Communication Assessment Tool: Content validity, test-retest reliability, interrater agreement, and implications for further development

### Topic: Assessment

#### Anh Nguyet Diep^1^, Jean-Christophe Servotte^1^, Nadia Dardenne^1^, Sophie Vanbelle^2^, Vanessa Wauthier^3^, Méryl Hajaoui^1^, Jonathan Goffoy^1^, Anne-Francoise Donneau^1^, Alexandre Ghuysen^1^

##### ^1^University of Liège, Belgium; ^2^Maastricht University, The Netherlands; ^3^Centre Hospitalier Régional de Namur, Belgium

###### **Correspondence:** Anh Nguyet Diep (anhnguyet.diep@uliege.be)

**Introduction**

The assessment of the communication skills of nurses in clinical settings bears both practical and educational significance. Despite the significance of communication skills to realize high-quality patient-care, the development and validation of such an instrument applicable in different cultural contexts is still lacking and faces certain challenges.

Additionally, the assessment of nurse-patient communication, which is dynamic and context-bounded, requires a fine-grained instrument, while not compromising the complexities of the skills under assessment.

The aim of the present study was to test a French-language version of the Health Communication Assessment Tool (HCAT), on a French-speaking sample in Belgium against different measures of content relevance, validity, and reliability. Based on cognitive fluency perspective, we developed three simulated scenarios registering three levels of skill performance, including low, medium, and high demonstration. Thus, the psychometric properties of the HCAT instrument can be thoroughly examined.

**Methods**

Ten experts in communication and 52 nurse educators participated in the study. Firstly, the instrument was translated from English to French using back-translation technique. After semantic equivalence was agreed upon, the experts rated the relevance of the 22 items making up the HCAT, resulting in the removal of one culturally-irrelevant item.

Subsequently, the experts and nurse educators rated the communication skills of the nurse in three scenarios. To ensure test-retest reliability, the nurse educators rated the scenarios twice within a four-week interval. The experts’ ratings were used as the gold standard when examining the reliability of the nurse educators’ ratings.

**Results & Discussion**

Exploratory and confirmatory factor analyses showed that the instrument could distinguish three aspects of communication skills, i.e. professional presentation, empathy, and trust building. Two-way random intraclass correlation coefficients (ICCs), ranging from 0.917 to 0.982, revealed that interrater agreement was excellent for consistency and absolute average-measures. However, at absolute single-measure, an ICC=0.425 suggested adequate interrater reliability for the medium-performed scenario, which decreased in low-performed (ICC=0.348) and high-performed (ICC=0.176) scenarios. Further, more than half of the items displayed poor test-retest reliability.

**Ethic Statement**

The authors declare that they have followed the guidelines for scientific integrity and professional ethics. Informed consent was obtained from all participants included in the study.

## A4 National Evaluation of a Low-Dose, High-Frequency Cardiac Resuscitation Quality Improvement Programme in the United Kingdom – CPR performance preliminary findings

### Topic: Assessment

#### Montana Mullen^1^, Catherine Fulwood^1^, Todd P Chang^2^, James Fenwick^3^, Victoria Withey^4^, Rod McIntosh^5^, Ralph James MacKinnon^6^

##### ^1^Manchester University NHS Foundation Trust, UK; ^2^Children’s Hospital of Los Angeles, USA; ^3^Basildon and Thurrock University Hospitals NHS Foundation Trust, UK; ^4^Spire Cheshire Hospital, UK; ^5^Borders General Hospital, Borders NHS, Scotland; ^6^Royal Manchester Children's Hospital, UK

###### **Correspondence:** Ralph James MacKinnon (ralph.mackinnon@mft.nhs.uk)

**Introduction**

Worldwide, there are over 135 million cardiovascular deaths each year, and the prevalence of coronary heart disease is increasing. Cardiopulmonary resuscitation (CPR) is a lifesaving intervention and the survival from cardiac arrest depends on early recognition of the event and also the immediate activation of the emergency response system. High quality CPR has been shown to save lives and has been identified as the “primary component in influencing survival from cardiac arrest”. To improve the outcome of cardiac arrest survival, a programme has been developed to provide users with simulation training and learning technology, delivering live feedback and on-going assessments. The UK RQI Programme has been designed to improve resuscitation education for healthcare providers with mandated quarterly CPR training on a specially designed cart in the workplace. The national evaluation aims to explore any impact of RQI on CPR performance, compliance, cost-savings and acceptability to end-users.

**Methods**

CPR performance data is under evaluation from five hospitals based in the United Kingdom. Hospitals entering the study complete an un-coached baseline assessment followed by quarterly coached training and a final un-coached assessment. Preliminary quantitative data has been evaluated for CPR performance in two hospitals. Initial change analyses have been conducted on three performance variables (ventilation only, compression only, rescuer CPR) for adult and infant CPR using t-tests. To date N=170 for adult CPR and n=156 for infant CPR.

**Results & Discussion**

The mean adult CPR baseline score of 46.56 (Standard Error of Mean=1.73) increased to 63.72 (SEM=2.54) (p=0.032), mean adult ventilation score of 21.89 (SEM=8.13) increased to 63.08 (SEM=2.64) (p=0.029), while the mean adult compression score increased from 51.13 (SEM=1.73) to 70.62 (SEM=2.54) (p=0.061). At this stage of the ongoing study, no significant differences in scores were seen in the infant skills across all three performance variables.

Preliminary findings suggest that the RQI programme, with quarterly access to a training cart in the workplace, is a useful tool to improve adult CPR performance. The ongoing study will shed further light on the effectiveness for RQI for both adult and infant CPR training in the UK.

**Ethic Statement**

This evaluation study has been approved and sponsored by the local research site (Manchester University Foundation Trust).

## A5 3D-printing soft materials to mitigate the effects of COVID-19 in medical education

### Topic: COVID- 19

#### Laszlo Jaksa, Andrea Lorenz

##### Austrian Center for Medical Innovation and Technology, Austria

###### **Correspondence:** Laszlo Jaksa (laszlo.jaksa@acmit.at)

**Introduction**

While the use of cadavers has a long history in medical education, their availability is affected by the diverse restrictions related to the COVID-19 pandemic. Moreover, the pandemic has made distance education more popular. Such trends in hands-on medical education call for easily transportable physical practice specimens. To simulate the human body, artificial anatomic models offer a viable alternative to cadavers. Such models are usually mass-produced, but in certain cases, patient-specificity is required. 3D-printing is a promising approach for producing models based on individual medical images. However, most technologies use hard materials, limiting the realism of the model. Printing with soft materials could bring higher simulation accuracy. Such improved models could improve hands-on medical education. In this research, we investigate the usage of a 3D-printing system based on the deposition of soft silicone rubbers, that can potentially print more realistic anatomic models.

**Methods**

**Fig. 2 (abstract A5).**
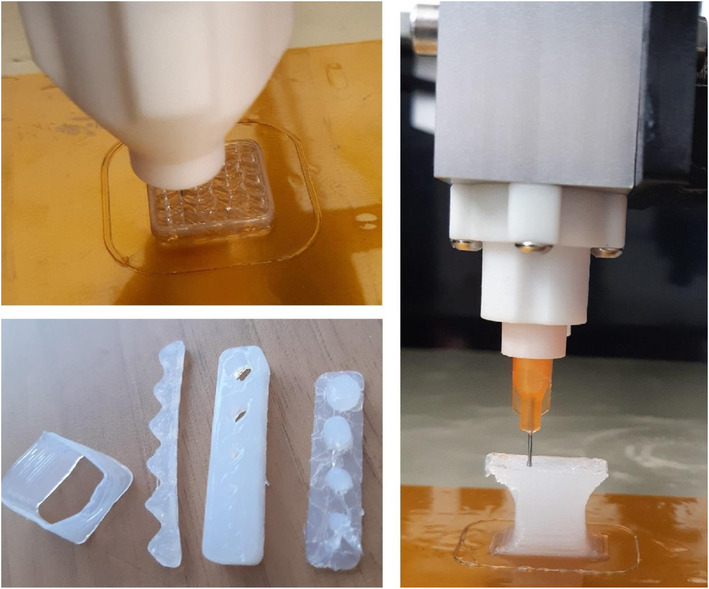
Printed objects

The system consists of a 3D-printer using a miniature screw extruder as printhead. Two commercial single-component liquid silicone rubbers were tested. Printed objects included various features like shells, corners, grooves, holes, columns, overhangs, and bridges (Figure 2). The limits of geometric freedom were determined via the qualitative evaluation of the printed features. Finally, to demonstrate the ability to modify the macroscopic properties of the bulk material, internal cavities are also made through infill structuring.

**Results & Discussion**

Both silicones were printable with a speed comparable to filament-based hard plastic printing. The minimal corner radius was 0.5 mm, the minimal groove width was 1 mm, the minimal column diameter was 3 mm and the maximal overhang angle was 60° for both materials. Further tests were conducted with both materials, showing a maximal bridge length of 2 mm, a maximal column aspect ratio of 2, and a maximal air volume fraction of 60% using gyroid infill structuring. Various air volume fractions resulted in palpable differences in mechanical properties. The results show that our system is capable of printing objects of soft material with high geometric freedom, including the abilityof tuning mechanical properties through infill structuring. With such a system, there is an opportunity to improve the hands-on simulation of the human body even in times when traditional means are restricted.

**Acknowledgements**

The presented work is funded by the Government of Lower Austria through the Austrian Institute of Medical Innovation and Technology.

**Ethic Statement**

The authors declare that they have followed the guidelines for scientific integrity and professional ethics. The article does not contain any studies with human or animal subjects.

## A6 Acute Frailty Simulation

### Topic: Curriculum Development

#### Peter Springbett

##### King’s College London NHS Foundation Trust, UK

###### **Correspondence:** Peter Springbett (peter.springbett@nhs.net)

**Introduction**

The care of frail, elderly patients poses specific challenges owing to their complex needs, multiple co-morbidities, polypharmacy and atypical presentations. Such patients form an increasing proportion of emergency department attendances and acute medical admissions, therefore providing relevant, specialist training to those who care for them is more important than ever.

With simulation firmly established as a core component of medical education, including geriatrics training, an acute frailty simulation course was created with the aim of increasing the confidence of junior doctors in providing care to frail patients, thereby improving their care in the acute setting.

**Methods**

A half-day simulation course was designed and delivered to junior doctors who care for acutely unwell frail, elderly patients. Scenarios were created to highlight specific learning points, with significant technical skills and human factors discussed during debriefing. Experienced faculty were recruited and high-fidelity mannequins used. Pre- and post-course questionnaires were completed by participants to assess their confidence in a number of domains related to the care of frail, elderly patients.

**Results & Discussion**

Confidence scores increased in all 8 domains after completing the course, with the highest percentage increase in ‘discussing and making end of life decisions’, ‘discussing DNAR’ and ‘assessing and managing acutely unwell frail, elderly patients’. Confidence also improved in leadership, teamwork and communication, but the percentage increase in these non-technical skills was slightly lower than the technical skills.

The increased confidence of junior doctors in managing acutely unwell frail patients suggests a positive impact of this course. The fact that human factors scores did not increase to the same extent as those of the technical skills may, at least in part, be as a result of pre-existing confidence in these areas being greater. Such non-technical skills are learned both informally through day-to-day experience, and formally on other simulation courses. Specific teaching on managing acute frailty is less frequently undertaken, therefore pre-course confidence levels in these domains were lower, with greater scope for improvement than in human factors.

Following the initial session, further dates are planned at both acute hospitals within the Trust at which modifications will be made based upon feedback received.

**Ethic Statement**

The authors declare that all procedures followed were in accordance with the ethical standards of the responsible committee on human experimentation (institutional and national) and with the Helsinki Declaration of 1975 (In its most recently amended version). Informed consent was obtained from all patients/participants included in the study.

## A7 Foundation Doctors Experiences and Attitudes to Patient Diversity in Simulation Based Medical Education

### Topic: Curriculum Development

#### Graham Picton, Alexandra Guy, Elizabeth Harrod, Michele Bossy

##### Royal Surrey County Hospital, UK

###### **Correspondence:** Graham Picton (graham.picton@gmail.com)

**Introduction**

Simulation has been described as a triad; the equipment, the environment and psychological components. Simulation-based medical education (SBME) should create a learning environment considering all three of these elements. At the last UK census, 83.5% of the population of Surrey described themselves as White British, leaving almost 1 in 5, which are not. We aimed to assess the previous experience of foundation doctors working at the Royal Surrey County Hospital (RSCH), establishing to what degree they had experienced attempts to recreate this diversity within SBME.

**Methods**

An electronic questionnaire was distributed to foundation doctors (doctors within their first 2 years post-qualification) assessing their previous experience of race, ethnicity and disability in SBME (assessed on a scale of 4 qualitative indicators: no, not at all, once, occasionally and frequently) and their views on whether replicating the diversity of their patients in SBME would improve the learning experience (assessed on a 4-point qualitative indicator scale; yes a lot, yes somewhat, not sure and no). Respondents were also asked how they described their own race/ethnicity describes themselves (according to the categories used in the 2011 national census) and if they had a disability.

**Results & Discussion**

Results were collected from 36 foundation doctors at RSCH. 75% of doctors had not previously been involved in SBME with a manikin of racial background other than white. 52% had not been involved in SBME with patients from an ethnic minority background. 86% had not experienced SBME with a disability (Table 2).

Responses were variable as to whether increasing racial diversity would improve the respondent’s learning experience (4 responded yes a lot, 10 yes, somewhat, 8 not sure, 13 no) and responses were also variable when asked if they thought increasing representation of ethnic minorities would improve the learning environment (7 yes a lot, 16 yes somewhat, 4 not sure, 9 no) but respondents appeared more positive about whether including disabilities would improve their learning experience (11 yes a lot, yes somewhat 17, not sure 4, no 4).

Fifty-five per-cent of respondents described themselves as White British. No respondents considered themselves to have a disability. Overall, foundation doctors within our cohort have a limited experience of patient diversity within SBME. Where our respondents feel that the use of manikins of different racial backgrounds may not affect their learning experience, they did feel that introducing disabilities would improve their learning experience within SBME.

**Ethic Statement**

The authors declare that they have followed the guidelines for scientific integrity and professional ethics. The article does not contain any studies with human or animal subjects.

**Table 2 (abstract A7).** Responses to question on junior doctors previous exposure diversity within simulation

	No, not at all	Once	Occasionally	Frequently
During simulation training that you have experienced outside of RSCH (e.g. previous trusts/med school /courses) have you come across simulation based teaching with manikins with racial background other than white?	27	1	7	1
During simulation training that you have experienced outside of RSCH (e.g. previous trusts/med school /courses) have you come across simulation based teaching with scenarios involving patients from ethnic minority background?	19	1	13	3
During simulation training that you have experienced outside of RSCH (e.g. previous trusts/med school /courses) have you come across simulation based teaching with simulated patients who have disabilities coincidental to those which are the focus of the clinical aspect of the scenario (e.g. the patient has pneumonia but happens to be deaf)?	31	2	3	0

## A8 How significant are insignificant results in simulation research? A call for precision education in optimising cognition

### Topic: Curriculum Development

#### Serkan Toy^1^, Christina Miller^1^, Adam Schiavi^1^, Rodrigo Daly Guris^2^

##### ^1^Johns Hopkins University School of Medicine, USA; ^2^Children’s Hospital of Philadelphia, USA

###### **Correspondence:** Serkan Toy (stoy1@jhmi.edu)

**Introduction**

Optimising learning for post-graduate medical trainees is challenging given the need to rapidly achieve competency in essential skills. We present secondary analyses from two separate randomized-controlled studies with negative results which could offer significant educational implications. In both studies, trainees improved targeted skills through simulations, however, experimental groups showed no difference. This study highlights the need for precision education using multimodal approaches allowing for data mining techniques in simulation research.

**Methods**

Both studies took place during a five-day simulation-boot-camp (SBC) designed for first-year anaesthesiology trainees, conducted over two consecutive years (2017 and 2018). Study-1 (S1) focused on speaking-up in the operating room, while Study-2 (S2) concerned managing intraoperative oxygen desaturation. Both studies included baseline and follow-up simulations. S1 had two post-test simulations and S2 included three during the SBC. Measures included a modified advocacy-inquiry rubric for speaking-up (S1) and a time-sensitive skills checklist for desaturation management (S2). NASA-TLX was used to measure cognitive load (CL).

A multivariate hierarchical cluster analysis was used to explore whether the combined sample in each study formed homogenous clusters beyond experimental grouping based on performance. We used mixed-design ANOVAs to examine differences between clusters on performance and CL. Using chi-square tests, we examined whether background variables were associated with cluster membership.

**Results & Discussion**

**Fig. 3 (abstract A8).**
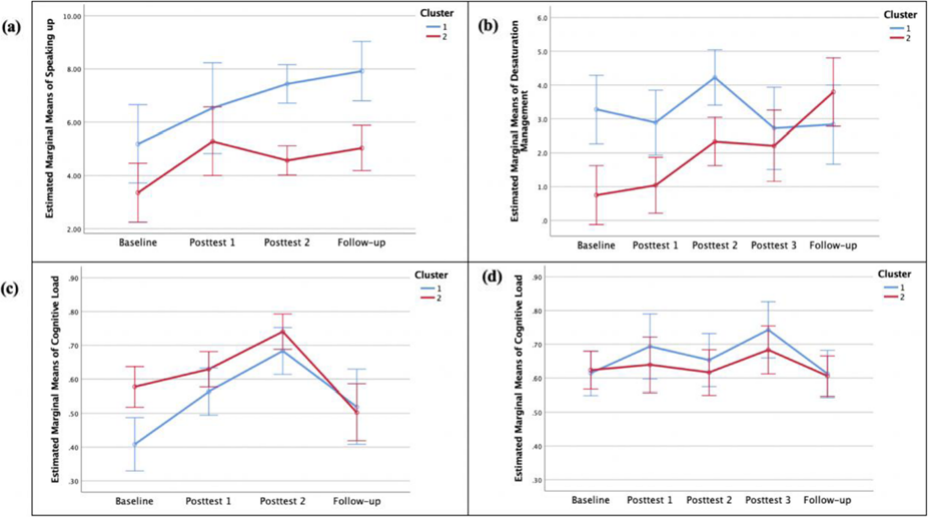
Line graphs showing clusters by (a) speaking-up scores (out of 10) in study 1, (b) desaturation management scores (out of 6) in study 2, (c) cognitive load, scaled NASA-TLX, in study 1, (d) cognitive load, scaled NASA TLX in study 2. Error bars indicate 95% confident intervals

Twenty-two trainees were included in S1 and 21 in S2. In each study, a two-cluster solution emerged. In S1, Cluster 1 (n= 8) consistently outperformed Cluster 2 (n = 14) (Fig.3a). These differences were significant at the second post-test and follow-up simulations (p < 0.001). Similarly, Cluster 1 (n = 9) in S2 outperformed Cluster 2 (n = 12) at baseline and first two post-tests (p < 0.005) (Fig.3b). Cluster 1 in S1 reported lower CL overall (Fig.3c). Clusters in S2 did not show any CL differences (Fig.3d). No associations were found between cluster membership and experimental groups, gender, age, prior training, or prior simulation exposure in either study.

Performance differences between clusters seem to persist over time for a non-technical (communication) skill, while they seem to disappear for a technical skill. We were not able to identify explanatory variables for these distinct clusters.

Future studies could examine additional variables, i.e. personality profiles and physiological data. These results emphasise that not all individuals benefit from simulation in the same manner. The timing of simulation activities and the nature of the targeted skills may play a role in this variability.

**Ethic Statement**

The authors declare that all procedures followed were in accordance with the ethical standards of the responsible committee on human experimentation (institutional and national) and with the Helsinki Declaration of 1975 (In its most recently amended version). Verbal informed consent was obtained from all participants included in the study.

## A9 Building a rubric for peer guidance on feedback, debriefing and conflict management: Delphi method in an expert community of practice

### Topic: Debriefing

#### Clement Buléon^1^, Demian Szyld^2^, Walter Eppich^3^, Mary Fey^2^, Janice Palaganas^2^, Robert Simon^2^, Jenny Rudolph^2^

##### ^1^Normandie Univ, UNICAEN, CHU de Caen Normandie, Pole Réanimations-Anesthésie, NorSimS, 14000 Caen, France; ^2^Center for Medical Simulation, Massachusetts General Hospital, Harvard Medical School, USA; ^3^Departments of Pediatrics and Medical Education, Northwestern University Feinberg School of Medicine, USA

###### **Correspondence:** Clement Buléon (clement.buleon@wanadoo.fr)

**Introduction**

Feedback and debriefing benefit from sharing one’s own perspective and goals, eliciting those of others and finding a way to collaborate, and learn across differences. Evidence-based scripts build skills, but scarce expert time, ineffective peer feedback, and lack of precision on how to assess or improve “bite-sized” chunks of conversation inhibit rapid skill-building of these crucial skills.

Breaking conversations down into blocks supports skill-building. We propose a multi-use conversational “Advocacy Inquiry Molecule” (AIM) and a rubric to assess it. Such a rubric can facilitate rigorous peer guidance for in-person or distributed on-line communities of practice.

We drew on existing research to develop a AIM for conversations that benefit from explicit signposting of topics, sharing one’s own perspective, learning other people’s, and bridging those perspectives to support collaboration or learning. It includes 5 successive components:

1. Preview: Signposts the topic of the conversation.
2. Observation: Describes concrete actions seen or statements heard.
3. Point of View: compares the feedback giver’s observation to a given standard and describes the implications.
4. Inquiry: Is an open-ended question and explores the receivers’ perspectives.
5. Listening: Hearing, acknowledging, and inviting receivers’ to share perspectives.

**Methods**

This Delphi method study built a consensus behaviorally anchored rating scale (BARS) based on the 5 elements of the AIM. Conducted in 4 anonymous survey rounds (R1-4) with research ethics approval, the study canvassed 39 international experts. They described “good” and “poor” behaviors for each element (R1); rated behaviors clumped into thematically alike descriptors according to importance (R2); selected the 6 most important (R3); classified them according to degree of difficulty to master (R4). Elements are rated with a seven-point scale: “Ineffective” (1) to “Extremely effective” (7).

**Results & Discussion**

In R1, investigators coded 871 raw descriptors into 118 like terms to provide the first set of good and poor descriptors for the 5 elements. R2 ranked descriptors and R3 selected 6 descriptors for each element. Bottom ranked items were discarded. Agreement on kept descriptors was high (87 to 100% per descriptor), agreement on discarded ones was lower (56 to 91% per descriptor). In R4, descriptors described were categorized from beginner to advanced (higher score). Final descriptors and rank order were used to create BARS (element “Preview” in Table 3).

The rubric built through 4 rounds of Delphi study could lower barriers to mastering key conversation skills. It can be used to spur peers to practice and provide each other feedback on defined “bite-sized” chunks of conversation.

**Acknowledgements**

For the IRIS (International Research and Innovation Simulation) group: Al Zariagi U, Almarshed A, Barlow M, Belle A, Buléon C, Cheng A, Del Moral I, Diederich E, Eller S, Eppich W, Fey M, Gardner R, Ghuysen S, Kardon Edgren S, Kolbe M, Lenes A, Janssens S, Maestre J, Minehart R, Morse K, Mullen A, Muller S, Ng G, Ortiz G, Palaganas J, Peterson DT, Raemer D, Reedy G, Rock L, Rubio R, Rudolph J, Rudolph M, Simon R, So HY, Sopka S, Szyld D, VanDijk J, Weller J,.Yager P.

**Ethic Statement**

The authors declare that they have followed the guidelines for scientific integrity and professional ethics. The article does not contain any studies with human or animal subjects.

**Table 3 (abstract A9).** Example of BARS for the FM element “Preview”

Preview						
1	2	3	4	5	6	7
Ineffective	Slightly Ineffective	Slightly Effective	Mostly Effective	Effective	Very Effective	Extremely Effective
· Off-putting words, threatening language · No preview or signal of topic change · Points out or “calls out” individuals in an unwelcome way · Misleading preview · Includes assumptions or inferences · Includes a judgment (may be hidden), or an assessment of performance			· Orients the listener to topic/Describes the topic/Signals a change of topic · Uses simple, clear terms appropriate to listeners · Is a neutral statement, does not evaluate performance · Specific: might address who/what/when/where · Seeks permission/Invites to discuss · Is parsimonious, as concise as possible			

## A10 Debriefing in operating theatre areas using the TALK© Framework

### Topic: Debriefing

#### Iago Enjo-Perez^1^, Esther Leon-Castelao^1^, Andrew Hadfield^2^, Pedro Castro-Rebollo^3^, Cristina Diaz-Navarro^2^

##### ^1^University of Barcelona, Spain; ^2^University Hospital of Wales, UK; ^3^Hospital Clinic, Spain

###### **Correspondence:** Iago Enjo-Perez (enjo@ub.edu)

**Introduction**

The “Five Steps for Safer Surgery” (5SfSS) document sets the current standards for patient safety in operating theatre environments in the United Kingdom. Debriefing, the process of an individual or team formally reflecting on their performance after a particular task, a shift or a critical event, is the fifth step in 5SfSS. However, it is not widely embedded in routine theatre behaviours yet.

The TALK framework is a simple and practical approach to structured feedback and team self- debriefing. It facilitates reflection and guides a short, constructive and non-judgmental dialogue after a case or clinical session whenever new insights might be learnt.

The aim of this study is to analyse the impact of introducing the TALK framework in compliance with 5SfSS in a university hospital. This study is part of a wider TALK research and implementation project funded by the European Commission under a Horizon 2020 MCSA-RISE grant.

**Methods**

This is an interventional study, carried out in short surgical stay and main theatre areas in the University Hospital of Wales. Elective lists that discharged patients to post-anaesthetic recovery areas were included. Emergency and cardiac lists were excluded.

The TALK structure was offered to operating theatre staff as a guide for clinical debriefing through a simple intervention which included short training sessions, departmental presentations and reflective discussions with multi-professional theatre staff. Debriefing was encouraged for those occasions in which team members considered it necessary.

Data collection included compliance with 5SfSS, consideration, practice and details of debriefing. Baseline and follow-up data (6,12,18 months) were collected. Ethical approval and informed consent were obtained.

**Results & Discussion**

In the 460 theatre lists included, there was consistently high compliance with team briefing and WHO checklist (≥97%). Consideration of debriefing increased significantly immediately after intervention and this difference continued at 18 months (36% at baseline vs 60% at 18 months, p=0.003). Practice of debriefing also increased significantly by 6 months, but then declined (23% baseline, 39% at 6 months, 30% at 18 months). We analysed the reasons for not debriefing when the teams had considered performing it: in 81% of cases they reported “no issues to debrief”.

These data suggest an initial cultural change, although reinforcement interventions are needed in order to maintain debriefing consideration and performance, and especially to identify potential themes for debriefing, in particular regarding positive performances.

**Ethic Statement**

The authors declare that they have followed the guidelines for scientific integrity and professional ethics. Ethical approval at University Hospital of Wales and informed consent were obtained.

## A11 Shining a light on debriefing: what actually happens in rural simulation-based education

### Topic: Debriefing

#### Kirsty Freeman, Sandra Carr, Colleen Fisher

##### University of Western Australia, Australia

###### **Correspondence:** Kirsty Freeman (kirsty.freeman@uwa.edu.au)

**Introduction**

Rural doctors are thrust into the role of providing simulation-based education without any training. A simulation activity is made up of several components, including a debrief. The debriefing component is essential for the transfer of knowledge, skills and attitudes to the clinical environment, ultimately improving future performance. Despite the importance of the debriefing component of the simulation activity, little is known about how rural educators conduct this debriefing.

This paper describes the experiences of rural doctors who facilitate debriefing during simulation-based education in rural and remote environments.

**Methods**

This study employed a mixed methods sequential explanatory design. The first phase of the study involved the collection of demographic data through an online survey. Purposeful sampling was used to select participants for the second phase in which participants were observed whilst conducting a debrief. The Debriefing Assessment for Simulation in Healthcare (DASH) tool was used to measure the effectiveness of the debrief from the perspectives of the learner, the debriefer, and the researcher. Semi-structured interviews were then used to explore the experience of the rural doctors in facilitating the debrief.

**Results & Discussion**

**Fig. 4 (abstract A11).**
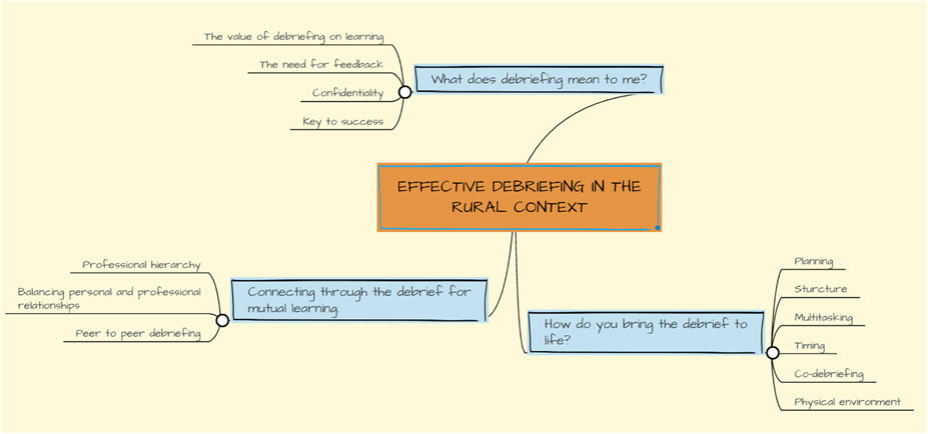
Identified key themes

In relation to the collection of demographic data 21 rural doctors responded to the electronic survey. The results showed that the average rural medical educator in Western Australia is male, aged between 40-49 years, completed medical training in Australia, and is working as a consultant in an emergency department. Analysis of the DASH scores showed a statistically significant difference between debriefer and learner groups across elements two to six. There was no statistically significant differences between the researcher and debriefer group. Qualitative analysis identified three key themes (Figure 4): (1) the meaning of debriefing; (2) bringing the debrief to life; and (3) connecting through the debrief for mutual learning.

Whilst the experience of facilitating the debriefing component of simulation-based education in rural and remote environments presents challenges, this study highlights the positive impact of the relationship between debriefer and learner, suggesting that the learning conversation does not and should not conclude at the end of the debrief, but continue to other clinical skills and bedside teaching opportunities.

**Ethic Statement**

Ethical approval was granted from the University of Western Australia Human Research Ethics Committees, reference number RA/4/1/7102. For data to be collected within regional hospitals approval was also granted by the Western Australian Country Health Services Human Research Ethics Committees (reference number 2014:22), and the Kimberley Aboriginal Health Planning Forum Research Subcommittee (reference Project 2014-015), a requirement for any research data collected in the Kimberly region of Western Australia.

## A12 The use of simulation debrief to further drug safety education in anaesthesia

### Topic: Debriefing

#### Michael Ma^1^, Robert Owens^1^, Crina Burlacu^2^, Osmond Morris^3^, Deirdre McCoy^1^

##### ^1^Royal Victoria Eye and Ear Hospital, Ireland; ^2^St. Vincent's Hospital, Ireland; ^3^St. James’s Hospital, Ireland

###### **Correspondence:** Michael Ma (mikelma@gmail.com)

**Introduction**

Medication errors (ME) continue to plague modern health care. It is estimated that ME cost the NHS £1.6 billion, contributing to 22,300 deaths annually. Medication related claims in Ireland estimate a median annual cost of ME to be €60,991. Between 2011 and 2016, 13% of ME leading to claims were identified as general anaesthetic medications. In the perioperative setting the anaesthetist is solely responsible for all aspects of medication management. These responsibilities include prescribing, preparing, and administering each medication without assistance, and often under highly stressful circumstances in a time critical manner. The intraoperative ME rate is known to be as high as 5%. In our study, simulation based learning was used to increase awareness and educate on the dangers of anaesthetic related ME. Our aim was to evaluate retention of key learning points by novice anaesthetic trainees who had observed simulated medication errors.

**Methods**

Novice anaesthetic trainees observed five high-fidelity simulation scenarios of commonly occurring perioperative ME. Through debriefing we placed a particular emphasis on key learning points relating to both technical and non-technical skills. Our primary outcome measure was the rate of recall of critical key messages at two weeks after the initial exposure. Candidates were also questioned about an ME awareness, change in practice, application of concepts, episodes of ME and an increased willingness to report drug errors.

**Results & Discussion**

Our preliminary data shows a recall rate of technical skills of 38.8%, and non-technical skills of 80%. The overall recall rate at 2 weeks was 52.5%. 100% of candidates reported increased ME awareness, 50% had changed their practice, application of key messages in 62.5%, and a 75% increase in likelihood to report drug errors. These results show a predominance in recall of non-technical skills, factors which are known to contribute significantly to ME. These initial findings, in our view, reflect a beneficial effect of simulation debrief on recall and on medication safety.

**Ethic Statement**

The authors declare that all procedures followed were in accordance with the ethical standards of the responsible committee on human experimentation (institutional and national) and with the Helsinki Declaration of 1975 (In its most recently amended version). Informed consent was obtained from all patients/participants included in the study. All institutional and national guidelines for the care and use of laboratory animals were followed.

## A13 A whirlwind of emotions: Medical students lived experience of emotions in simulation

### Topic: Innovation

#### Gerry Gormley^1^, Diana Dolmans^2^, Erik Dressen^2^, Claudia Behrens^3^

##### ^1^Queen’s University Belfast, Northern Ireland; ^2^Maastricht University, The Netherlands; ^3^Universidad Católica del Norte, Chile

###### **Correspondence:** Gerry Gormley (g.gormley@qub.ac.uk)

**Introduction**

Simulation based education (SBE) affords learners opportunities to develop their clinical skills and behaviours. By extending learners beyond their comfort zone, SBE can be highly emotive and induce intense emotions. Such emotional states have potential to influence learning. Moreover, SBE has potential to induce negative emotions and stress reactions. Despite the importance of emotions within simulation, there is limited empirical evidence of the interplay between emotions, performance and learning – especially experienced ‘in-the-moment’ by learners. Unearthing greater insights into these emotional states - has potential to guide how best we manage such emotions, whilst optimising learning. This study aimed to provide a deep understanding of the lived experience of medical students’ emotions within simulation, and their impact on performance and learning.

**Methods**

Consenting final-year medical students were recruited and sampled purposefully. Wearing video-glasses, eight participants took part in a ward-based simulation exercise that focused on managing acutely unwell patients.

Point-of-view (PoV) video-footage was used to elicitate exploratory interviews, which were transcribed verbatim. Using a Hermeneutic Phenomenological approach, all transcripts were analysed using Template Analysis reflexively.

**Results & Discussion**

Analysis yielded four main themes about individual’s emotional states in simulation and factors that shape these emotions: ‘Nervous anticipation’; ‘Shock and awe’; ‘In the moment: Flowing or buffeting with the emotions’ and ‘Safe-landing?’

PoV video-footage helped to elicitate unique insights into the complex tacit and embodied emotional dynamics that occur within simulation. Despite the controlled nature of SBE, student’s emotional responses are dynamic, often unpredictable and at times fragile. Often the tone for the emotions was set just prior to the simulation. Student’s emotions were closely entwined with their perceived performance and how this aligned to expectations of their future professional-self.

Negative and positive emotions could be experienced in duality – with a fine balance adaptive and maladaptive emotional regulation. If students experienced their performance to have met desired standards, more positive achievement emotions were induced and a greater receptive state for learning. However if they experienced their performance to have fallen short of expected standards, this could promote more negative emotions. In so doing, SBE challenging their professional identity and hinder learning. Negative emotions could drive a negative loop of reactions/counter-reactions between emotions and performance. The emotional residue from which could persist, even despite debriefing. Our findings provide a foundation to guide educators in managing the emotional climate within simulation to optimise learning.

**Ethic Statement**

The authors declare that all procedures followed were in accordance with the ethical standards of the responsible committee on human experimentation (institutional and national) and with the Helsinki Declaration of 1975 (In its most recently amended version). Informed consent was obtained from all patients/participants included in the study. All institutional and national guidelines for the care and use of laboratory animals were followed.

## A14 Ethnographic Study of Health Personnels Use of Human Factors Skills in Teamwork

### Topic: Interprofessional/Team Education

#### Lotte Abildgren

##### Odense University Hospital, Denmark

###### **Correspondence:** Lotte Abildgren (ouh.simc@rsyd.dk)

**Introduction**

Simulation is increasingly used to train the postgraduate healthcare personnel in Denmark, e.g. in relation to improving teamwork. Research shows that 70-80% of all adverse events can be traced back to a defect in personnel’s human factor skills, such as communication, teamwork and decision making. The prevention of these adverse events is most often met by applying theoretical knowledge or clinical instructions on sub-elements of the complex everyday practice, which can be difficult to alter into new action skills and routines. The hypothesis is that ethnography together with training and debriefing can explore what kind of learning the personnel transfer from training to daily practice. The purpose of the underlying study is to develop basic, interdisciplinary knowledge of how healthcare personnel’s transfer human factor skills from simulation-based training to action in the complex clinical practice.

**Methods**

Through the use of cognitive ethnography transfer of knowledge were investigated. Video data of healthcare personnel’s use of human factor skills in teamwork before, during and after participation in an in situ simulation training course were collected. Data were collected in the four different units, at two different hospitals in the Region of Southern Denmark. Data consist of video, field notes, informal interviews and questionnaires.

**Results & Discussion**

The result shows that ethnography explores the field of simulation. It is possible to look upon both the educators and the participant actions and furthermore to look on the dynamics within the teams in the everyday, the simulations and debriefings. The extension of the methodologic strategies for research in simulation can broaden the knowledge of how and what the participants learn in a larger scale than e.g. pre- and post-tests. The analysis of all the data of the underlying project is still not finished, therefore it is not possible to say if and how transfer is done, but I would like to discuss the use of cognitive ethnography to explore the results and possible transfer of simulation based training.

Implications for practice: Using cognitive ethnography to explore transfer of simulation-based training can result in: opportunities to develop a generic method of testing and implementing competent use of human factors and thereby increase patient safety; Knowledge on the development of personnel’s educational curricula in order to insure transfer of knowledge into performance in clinical practice; And developing the basis of a methodological approach capable of evaluating NTS transfer from in situ simulation training to clinical practice.

**Acknowledgements**

OPEN, Odense Patient data Explorative Network, Odense University Hospital, Odense, Denmark, sdu.dk/ki/open. Anaestesiology-Critical Care Unit & Unit of infectious diseases, OUH, Odense University Hospital Critical care unit & Emergency unit, SHS, Hospital of Southern Denmark.

**Ethic Statement**

The authors declare that they have followed the guidelines for scientific integrity and all procedures followed were in accordance with the ethical standards of the responsible committee on human experimentation (institutional and national) and with the Helsinki Declaration of 1975 (In its most recently amended version). Informed consent was obtained from all patients/participants included in the study.

## A15 Home-made pediatric severe burn moulages: Recipes, and impact on team members in in-situ simulations

### Topic: Interprofessional/Team Education

#### Senay Sarmasoglu^1^, Nazmiye Celik^2^, Melih Elcin^1^, Emrah Senel^3^

##### ^1^Department of Simulation in Healthcare, Hacettepe University, Turkey; ^2^Department of Pediatric Burn Center, University of Health Sciences, Turkey; ^3^Department of Pediatric Surgery, Ankara Yildirim Beyazit University, Turkey

###### **Correspondence:** Senay Sarmasoglu (senay.sarmasoglu@hacettepe.edu.tr)

**Introduction**

One of the ways to increase involvement in simulation is to increase the realism of the simulation. Moulage enhances the authenticity of the simulation and contributes to the participants’ better interaction between the clinical case and the simulation, guiding the participants to make the exact diagnosis and provide appropriate treatment. The aim of this study is to reveal the evaluations of experienced burn-team members about the fidelity, case management and their participation in the scenarios of three different burn types, which are frequently encountered in real life pediatric burn patients.

**Methods**

This study was a part of a research project conducted at the Children's Hospital Burn Center in Ankara, Turkey. In the cross-sectional study, the moulages applied on manikins were evaluated by nine experienced pediatric burn team members (nurses and physicians) using a 5-point Likert-scale during in-situ simulations. Descriptive statistics were used in the analysis of the data.

**Results & Discussion**

The mean age of the health professionals was 36.73 ± 7,862 and their experience in the related unit ranged from 6 to 23 years (mean ± ss = 7.3 ± 7.2). The health professionals found that the moulages were realistic in all the cases.

Moulages led the burn team members feel as if they were real burn cases, facilitated their involvement in the scenario, and had positive impact on their case management. The burn-team stated that cost effective burn moulages prepared at home can be used as an effective tool in the education of health professionals working in the field of burn and the students studying in the field of health sciences. In simulation applications, it is thought that burn moulages will be useful in order to create an climate of fidelity, and to enable the participants actively engaging in the simulation process.

**Ethic Statement**

The authors declare that they have followed the guidelines for scientific integrity and professional ethics. The article does not contain any studies with human or animal subjects.

## A16 Simulation based improvement in team emergency reaction

### Topic: Interprofessional/Team Education

#### Olegs Sabelnikovs, Marina Sarkele

##### Department of Clinical Skills and Medical Technologies, Rīga Stradiņš University, Latvia

###### **Correspondence:** Marina Sarkele (marina.sarkele@rsu.lv)

**Introduction**

Employee training, emergency response drills identify deficiencies in emergency response and reduce incidents based on risk assessment during team simulations. Emergencies can cause significant downtime and production loss that affects patient safety. In situ simulations has a great impact on improvement of interaction among medical professionals. This benefits in better performance and increases patient safety.

The goal of the study is to implement in situ simulation training to increase level of team self confidence and shorten time of reaction to emergency case. To compare the results before and after training.

**Methods**

We observed 10 massive bleeding cases in Obstetric department during the last two months with delayed response and bigger blood loss as expected. We performed in situ simulation training drills among personnel to increase the level of response. There were trained teams of physicians and midwifes. After training we observed 10 cases of massive bleeding to compare the results. We were looking for time of all team activated, time of vital function monitoring, time of blood bank activating.

**Results & Discussion**

Before simulation training we found that average time of all team arrival including anesthetist is 486 sec (378- 713 sec). Vital signs monitoring were provided in 243 sec (189- 501 sec). Time of activating blood bank 785 sec (601- 1003 sec) in average. Personnel of the department mentioned stress and panic related time loss. After training time of reaction to emergency bleeding was significantly shortened. Monitoring were provided in 120 seconds before all team arrived and call to blood bank was given immediately after situational assessment in 420 sec.

Simulation of nearmiss cases can significantly improve the reaction of team through risk assessment and increase in level of confidence.

**Ethic Statement**

The authors declare that they have followed the guidelines for scientific integrity and professional ethics. The article does not contain any studies with human or animal subjects.

## A17 Theory-informed Simulation Design for Safer Paediatric Tracheostomy Care

### Topic: Interprofessional/Team Education

#### Frith Cull^1^, Rachel Imber, Colette Laws-Chapman^2^, Mary Lavelle^3^, Ranjev Kainth^2^

##### ^1^Guy’s and St Thomas’ NHS Foundation Trust, London, UK; ^2^ASPIH, UK; ^3^Mental Health in the School of Health Sciences at City, University of London, UK

###### **Correspondence:** Frith Cull (frith.cull@gstt.nhs.uk)

**Introduction**

Tracheostomies are life-preserving airway adjuncts used increasingly long-term in the paediatric population. Tracheostomy-related emergencies represent loss of a patent airway, necessitating immediate action. Regular training of healthcare professionals in routine and emergency care, with the inclusion of simulation training, is therefore essential.

**Methods**

**Fig. 5 (abstract A17).**
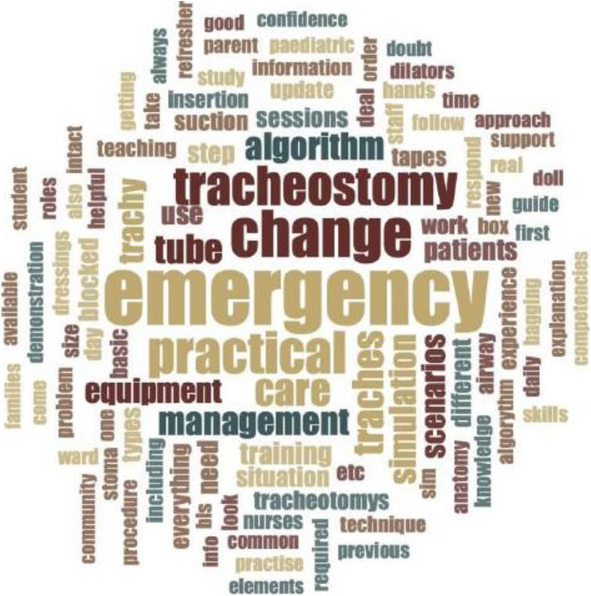
Word cloud of participants’ responses to learning needs analysis survey. Participants were asked “What should be included in a study day about emergency tracheostomy care for it to be a useful day for you?”

Local need mandated a structured approach in designing a new paediatric tracheostomy emergency care simulation course for healthcare professionals in a tertiary paediatric hospital in London. A multi-faceted learning needs analysis (LNA) was undertaken including a mixed-methods survey (n=285). Key findings included 58% unaware of the emergency algorithm with ~50% of these having been a first responder. Qualitative analysis of respondents perceived needs centred around tube changes, emergency management, algorithm knowledge and equipment (Figure 5). Through this LNA, relevant course objectives were defined to ensure constructive alignment.

There is call for the simulation community to consider educational theory in course design to maximise learning. Therefore, we designed didactic algorithm teaching, combined with ‘hands-on’ practical skill experience in six skill stations (such as tube change and suctioning) using Peyton’s four-stage teaching technique, allowing participants to move into the application stage of learning during two simulation scenarios. Structured debriefing (using the Diamond Debrief) maximises discussion of both technical elements and human factors discussion. Participants also experience rapid cycle deliberate practice in operationalising the algorithm prior to the simulation scenarios.

Pre- and post-course questionnaires are undertaken, using the Human Factors Skills for Healthcare Instrument (HuFSHI) and knowledge-based speciality specific questions to enable design-focused evaluation.

**Results & Discussion**

To date, the course has run nine times and reached 96 practitioners, including nurses, nursing assistants, doctors, paramedics, physiotherapists, speech and language therapists and clinical physiologists.

Fifty-five participants’ questionnaire data was complete and utilisable for paired analysis. Paired t-tests compared participants’ mean scores pre- and post-training. Following training, there was significant improvement in participants’ confidence in managing aspects of tracheostomy care (t(54)=16.95, p<.0001) and Human Factors Skills (t(54)=5.81, p<.0001). Chi squared analysis showed that significantly more participants were familiar with the emergency algorithm post training (Chi (1)=56.79, p<.001) and could correctly state blocked tube management post training (Chi (1)=10.29, p=.001).

This intervention with significant improvement in multiple domains, reinforces the position that simulation educational design should be theory-informed with constructive alignment enabling meaningful evaluation. Further outcomes will be presented from this learner-centred module, which aims to provide maximal benefit to patients from staff confident in tracheostomy care.

**Acknowledgements**

Samantha Gainfort, Suying Man & Victoria Thompson (Paediatric ENT Practice Development Nurses)

**Ethic Statement**

The authors declare that they have followed the guidelines for scientific integrity and professional ethics. The article uses learner questionnaire data which has been approved under a blanket approval in place for questionnaires undertaken as part of SaIL centre work.

## A18 Development of a mixed reality system for first-aid training

### Topic: New Technologies

#### Serena Ricci^1^, Elisa Girau^2^, Fabrizio Mura^1^, Maura Casadio^1^, Marco Chirico^1^, Fabio Solari^1^, Manuela Chessa^1^

##### ^1^University of Genova, Italy; ^2^CUNY School of Medicine, USA

###### **Correspondence:** Serena Ricci (serena.ricci@edu.unige.it)

**Introduction**

Healthcare simulation is part of medical training since it allows “learning by doing”. In other words, students can learn medical procedures in a riskless environment. Also, they learn how to interact with patients, as well as to deal with stressful situations. Currently, high-fidelity simulators are realistic tools responding to therapies and simulating different cases. However, the environment in which the simulation takes place may affect the experience of the student, eventually resulting in a low involvement. In this context, Virtual Reality (VR) may enhance the simulation experience, giving a higher degree of immersivity. However, VR applications lack of the physical part allowing to manually perform medical tasks. The goal of this study was to combine VR with a human body mannequin, into a mixed reality model.

Specifically, the student is immersed into a VR scene with the addition of the haptic feedback when the mannequin is touched.

**Methods**

**Fig. 6 (abstract A18).**
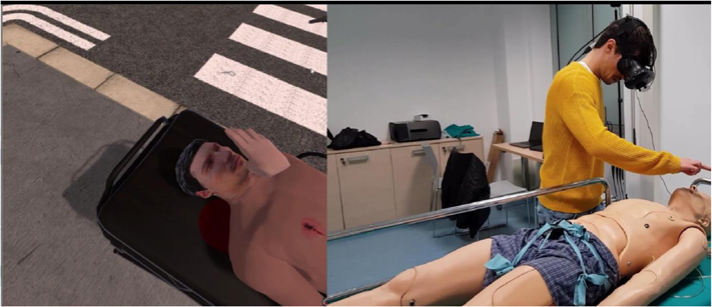
System consisting in a full-body mannequin, HTC Vive and Leap Motion

Our system consists in a full-body mannequin, HTC Vive and Leap Motion (Figure 6). The user wears the HTC Vive Head Mounted Display to be immersed in a VR environment, developed in Unity 3D and Blender. He can freely move in the scene, representing a city, and sees a representation of his own hands, thanks to the Leap Motion. Furthermore, he has to interact with an injured person who has a lung perforation and a gun shot (Figure 6). The user can stop both the asymmetrical breathing and the bleeding placing one hand on the wounds. Interactions between user and virtual patient give also haptic feedback due to the presence of the mannequin. Specifically, the mannequin is equipped with three Vive Trackers allowing for a real time correspondence between virtual and real (Figure 6).

**Results & Discussion**

The system has been tested in terms of accuracy and immersivity. In detail, we calculated the differences between mannequin and virtual representation on 30 points. Results indicate errors between 0.02 and 3.25cm, with the 75% of the points below 1.5n0.06 cm. User experience was evaluated on 27 subjects (age 20-30 years). Before and after a 10-minute simulation, participants filled out two questionnaires about cybersickness and user experience. No physical discomfort was detected after the simulation. Results on immersivity were positive: some users found the scene “videogame-like” but this did not affect the experience. Furthermore, the possibility to use the hands instead of the controllers helped with interaction, sense of presence and immersivity.

**Ethic Statement**

The authors declare that they have followed the guidelines for scientific integrity and professional ethics. The article does not contain any studies with human or animal subjects.

## A19 Effectiveness of the Checklist in the management of patients with major trauma: the CheLTS Study (Checklist in Trauma Simulation)

### Topic: Patient Safety

#### Valerio Stefanone, Irene Rasoini, Irene Tassinari, Francesca Innocenti, Rita Audisio, Federico Meo, Caterina Savinelli, Riccardo Pini

##### Azienda Ospedaliero-Universitaria Careggi, Italy

###### **Correspondence:** Francesca Innocenti (innocenti.fra66@gmail.com)

**Introduction**

Trauma is a leading cause of death and disability in the earliest decades of life. Management of major trauma is challenging for emergency physicians due to multiple, simultaneous and potentially fatal lesions. The aim of the present study was to test the effectiveness of a checklist in improving the management of patients with major trauma.

**Methods**

We tested our hypothesis in a simulation environment and we planned to include 25 teams; this is a preliminary analysis based on the performance of the first 12 teams. Our population included Emergency Medicine residents, divided in 4-member teams, in which the most expert was the team leader. We designed four scenarios, focused on the management of trauma. All the teams performed the four scenarios in a random sequence.

We created a checklist with the critical actions to be performed in trauma patients. We gave the checklist to all the teams alternatively during the first or the last two scenarios. The primary outcomes were the adherence to critical processes of care and the time to critical actions in the scenarios with versus those without the checklist.

**Results & Discussion**

We identified 52 critical actions, which had to be performed during the simulation and we compared the rate of performance in the scenarios with, compared to those without the availability of the check-list. Overall 5 actions were performed more frequently in the presence of the checklist: spine immobilization (33% without vs 100% with the checklist, p 0.002), screening for pneumothorax (13% without vs 88% with the checklist, p 0.002), screening for dorsal hemorrhages (54% without vs 100% with the checklist, p 0.043), evaluation of glycemia (20% without vs 80% with the checklist, p 0.022), and vitals re-evaluation (6% without vs 60% with the checklist, p 0.030). The time needed to perform the following actions was significantly reduced with the checklist: evaluation of refill time (03:15 with the checklist vs 07:00 without, p 0.047), evaluation of peripheral pulses (02:42 with the checklist vs 04:10 without, p 0.041), lab collection including blood cross-match (03:06 with the checklist vs 05:12 without, p 0.018), call for surgical consult (08:13 with the checklist vs 10:44 without, p 0.037).

In a high-fidelity simulation environment, the use of a checklist improved the promptness of relevant actions for the management of patients with major trauma. These findings suggest that checklists represent a valid tool to assist the care of these challenging patients.

**Ethic Statement**

The authors declare that all procedures followed were in accordance with the ethical standards of the responsible committee on human experimentation (institutional and national) and with the Helsinki Declaration of 1975 (In its most recently amended version). Informed consent was obtained from all patients/participants included in the study. All institutional and national guidelines for the care and use of laboratory animals were followed.

## A20 Improving quality health outcomes for patient using observational simulation methods

### Topic: Patient Safety

#### Patrea Andersen

##### University of the Sunshine Coast, Australia

###### **Correspondence:** Patrea Andersen (panders1@usc.edu.au)

**Introduction**

Working in partnership, a regional private hospital and university in Australia responded to accreditation audit findings by developing a series of simulation scenarios to enhance patient safety awareness. Framed within the Australian National Quality Health Service Standards simulations were captured on video, made into artefacts and deployed using observational simulation methods in annual mandatory professional education for Nurses and Allied Health staff during 2016 and 2018. The video artefacts captured a patient’s hospital Journey. Optimal and sub optimal versions of patient encounters including medication administration, infection control and prevention, patient identification and procedure matching, clinical handover and preventing falls were included.

**Methods**

Subsequent to ethical approval, a design-based research methodology including quantitative and qualitative data collection and analysis methods was employed to evaluate the initiative. A convenience sample (n=429) including Nurses (RN, EN), Nurse Assistants, Midwives, Physiotherapists, Occupational therapists and Anaesthetic Technical staff were recruited. A survey (Satisfaction with Simulation Experience Scale) and interviews were used to gather participant perceptions about the learning experience and its translation in practice. Quality data was mined to compare pre-reported patient safety quality data in relation to medication administration, infection control, falls and falls with injury with post intervention measures. Data for falls with injury included injury sustained as a result of the fall (e.g. skin tear, fracture, closed head injury). Standard statistical tests using SSPS (Descriptive, Chi Square, Pearson’s correlation, cross-tabulation and multivariant analysis) were employed for data analysis. Data comparison was based on per 1000 bed days. In Australia this is a commonly used statistic corresponding to the total number of days a bed was occupied during a month.

**Results & Discussion**

Satisfaction with the education programme ranked highly in evaluations. Participants believed that the initiative improved risk management, understanding of documentation requirements, and collaboration and communication amongst teams. Quality data highlighted the evidence of programme impact on patient health outcomes with a 73% decrease in falls with injury, 34% decrease in high alert medication error and 61% decrease in infection within 12 months. These results were sustained over the period of the study. Using observational simulation in this study promoted active engagement in professional development and has resulted in a change in practice culture which focuses on patient safety and reduced sentinel events. This presentation will report the project design and results evidencing the power of simulation methodologies in staff education and how this can translate in practice and improve patient safety.

**Ethic Statement**

The authors declare that they have followed the guidelines for scientific integrity and professional ethics. Ethical approval was given by both institutions involved in this research. Informed consent was obtained from all patients/participants included in the study. Participation was voluntary. Anonymity and confidentiality are maintained. Participants were informed that the research would be published and or presented at conference.

